# Effects of CO_2_ enrichment on photosynthesis, growth, and nitrogen metabolism of the seagrass *Zostera noltii*

**DOI:** 10.1002/ece3.333

**Published:** 2012-09-19

**Authors:** Ana Alexandre, João Silva, Pimchanok Buapet, Mats Björk, Rui Santos

**Affiliations:** 1 Marine Plant Ecology Research Group, CCMAR – Centre of Marine Sciences, Universidade do AlgarveFaro, Portugal; 2 Botany Department, Stockholm UniversityStockholm, Sweden

**Keywords:** CO _2_ enrichment, glutamine synthetase, growth, nitrate reductase, nitrogen uptake, photosynthesis, seagrasses

## Abstract

Seagrass ecosystems are expected to benefit from the global increase in CO_2_ in the ocean because the photosynthetic rate of these plants may be C_i_-limited at the current CO_2_ level. As well, it is expected that lower external pH will facilitate the nitrate uptake of seagrasses if nitrate is cotransported with H^+^ across the membrane as in terrestrial plants. Here, we investigate the effects of CO_2_ enrichment on both carbon and nitrogen metabolism of the seagrass *Zostera noltii* in a mesocosm experiment where plants were exposed for 5 months to two experimental CO_2_ concentrations (360 and 700 ppm). Both the maximum photosynthetic rate (P_m_) and photosynthetic efficiency (*α*) were higher (1.3- and 4.1-fold, respectively) in plants exposed to CO_2_-enriched conditions. On the other hand, no significant effects of CO_2_ enrichment on leaf growth rates were observed, probably due to nitrogen limitation as revealed by the low nitrogen content of leaves. The leaf ammonium uptake rate and glutamine synthetase activity were not significantly affected by increased CO_2_ concentrations. On the other hand, the leaf nitrate uptake rate of plants exposed to CO_2_-enriched conditions was fourfold lower than the uptake of plants exposed to current CO_2_ level, suggesting that in the seagrass *Z. noltii* nitrate is not cotransported with H^+^ as in terrestrial plants. In contrast, the activity of nitrate reductase was threefold higher in plant leaves grown at high-CO_2_ concentrations. Our results suggest that the global effects of CO_2_ on seagrass production may be spatially heterogeneous and depend on the specific nitrogen availability of each system. Under a CO_2_ increase scenario, the natural levels of nutrients will probably become limiting for *Z. noltii*. This potential limitation becomes more relevant because the expected positive effect of CO_2_ increase on nitrate uptake rate was not confirmed.

## Introduction

Projections that the current atmospheric CO_2_ concentration will double by the end of this century and that oceanic CO_2_ level will rise (IPCC [Intergovernmental Panel on Climate Change] 2007) have caused increasing interest in the research of the direct impacts of elevated CO_2_ on the marine environment (Gattuso et al. 1998; Feely et al. 2004; Guinotte and Fabry 2008; Hall-Spencer et al. 2008; Pörtner 2008; Porzio et al. 2011). It is expected that the seawater pH will decrease 0.3–0.4 units relative to present values before the year 2100 (Caldeira and Wickett 2003; Feely et al. 2004). The acidification of the seawater will induce changes in the carbonate chemistry, that is, in the relative proportions of the inorganic carbon species, carbon dioxide (CO_2_), bicarbonate (HCO_3_^−^), and carbonate (CO_3_^2−^), shifting the total dissolved inorganic carbon away from CO_3_^2−^ toward more HCO_3_^−^ and CO_2_ (Riebesell et al. 2007). This shift toward more HCO_3_^−^ is expected to benefit species that use it as a carbon source for photosynthesis in addition to CO_2_ (Beer et al. 2002; Mercado et al. 2003).

Seagrass-dominated ecosystems play an important role in the carbon cycle of coastal areas (Duarte and Chiscano 1999; Hemminga and Duarte 2000). The responses of seagrasses to elevated CO_2_ concentrations must be considered for an effective management of coastal regions in the future. Seagrass meadows are reported as one of the few ecosystems that may benefit from rising CO_2_ levels because their photosynthetic rates have been considered C_i_-limited at the current oceanic CO_2_ concentration (Beer and Koch 1996; Thom 1996; Zimmerman et al. 1997; Invers et al. 2001). Consequently, increases in seagrass production and growth may occur in a future high-CO_2_ scenario.

CO_2_ enrichment may also affect nitrogen uptake and the assimilation process, as growth enhancement at high-CO_2_ concentrations is expected to increase the nitrogen demand of plants (Stitt and Krapp 1999). In addition, the relative uptake rates of ammonium and nitrate may be altered by the acidification of the seawater resulting from CO_2_ enrichment, due to the involvement of protons (H^+^) in the nitrogen transport across the plasma membrane. In terrestrial plants, nitrate is cotransported with H^+^ across the membrane and consequently lower external pH facilitates nitrate uptake because of the higher H^+^ gradient outside the cell (e.g., Vessey et al. 1990). On the other hand, the lower external pH affects ammonium uptake because the higher content of H^+^ reduces the activity of H^+^-ATPase, which is involved in the cation transport into the cells (Marschner 1995). From the ionic balance perspective, lower pH levels in the seawater may reduce the ammonium uptake rates of seagrasses, whereas nitrate uptake rates may be unaffected or even increased.

The effects of CO_2_ enrichment on seagrasses have focused mainly on how elevated CO_2_ concentrations will affect seagrass productivity, light requirements, and nutrient content (Beer and Koch 1996; Thom 1996; Zimmerman et al. 1997; Palacios and Zimmerman 2007; Jiang et al. 2010). However, these effects were investigated with short-term (days) laboratory experiments, except the study of Palacios and Zimmerman (2007), in which experiments were run in outdoor aquaria for 1 year. Longer term studies are thus needed to account for the acclimation potential of seagrass species to increasing CO_2_. Furthermore, the response of seagrass nitrogen metabolism to CO_2_ enrichment is not known.

Here, we investigate the effects of CO_2_ enrichment on the carbon and nitrogen metabolism of the seagrass *Zostera noltii* in a mesocosm experiment where plants were exposed for 5 months to present (360 ppm) and future (700 ppm) seawater CO_2_ concentrations. We specifically aimed to assess the effects of CO_2_ enrichment on photosynthesis and growth, on the ammonium and nitrate uptake rates, and on the activity of nitrate reductase and glutamine synthetase, the two key enzymes of nitrogen assimilation. To the best of our knowledge, this is the first report on the effects of the global CO_2_ increase on the nitrogen metabolism of seagrasses.

## Methods

### Plant collection and experimental design

*Zostera noltii* is the most abundant seagrass species in Ria Formosa coastal lagoon, South Portugal (37°00′N, 7°58′W). The species develop along subtidal and intertidal areas and plays a major role in the lagoon's metabolism (Santos et al. 2004). In this system, the nutrient concentration in the water column is typically less than 5 *μ*M due to a high water exchange between the lagoon and the adjacent ocean in each tidal cycle. Ammonium and phosphate concentrations in the sediment porewater are higher (12–38 *μ*M for NH_4_^+^ and 2.5–14 *μ*M for PO_4_^−^), whereas the concentration of nitrate is almost negligible (<1 *μ*M) (Cabaço et al. 2008).

In order to preserve the integrity of the *Z. noltii* belowground plant parts and of its associated community, 20-cm diameter cores were carefully collected including plants and sediment, in March 2010. The cores were used to fill plastic boxes of 55 × 35 × 14 cm, which were placed in an outdoor mesocosm system at Centre of Marine Sciences (CCMAR) field station, near the donor meadow. The mesocosm consisted of two flow-through open systems running in parallel, one with seawater at the present CO_2_ concentration (360 ppm) and the other with twofold the present CO_2_ concentration (700 ppm), close to the “business as usual” scenario for 2100 of IPCC (Intergovernmental Panel on Climate Change) (2007) projections. Each system consisted of one head tank (1500 L) connected to two independent tanks (660 L each). Each of these tanks included four plastic boxes of *Z. noltii* and its associated community. Consequently, the experiment consisted of 2 CO_2_ levels × 2 replicates (660 L tanks), each bearing four plant units. The seawater used in the mesocosm was pumped from the lagoon into the head tanks after passing through a sand filter. The flow rate to each replicate unit was about 210 L/h. CO_2_ was bubbled into the head tanks from a CO_2_ tank to achieve the experimental CO_2_ concentrations (360 and 700 ppm). The rate of CO_2_ injection into the system was controlled by the pH level of the seawater using pH probes connected to CO_2_ controllers (EXAtx 450; Yokogawa, Tokyo, Japan). We acknowledge that this is a pseudoreplicated design, but the alternative option to control pCO_2_ individually in each tank would result in an added degree of error related to the difficulties of maintaining the same pCO_2_ values between tank replicates. The maintenance and control of elevated pCO_2_ levels in experimental tanks is not a straightforward process, but rather a difficult task, with countless small problems. Therefore, we considered that it was preferable to supply all the tanks with the same batch of water (and hence the same pCO_2_), even at the cost of falling into pseudoreplication. We trust that there is a high probability that the observed effects are due to the CO_2_ variable rather than to some undetected confounding effect between head tanks because the tanks were exactly the same size and type with exactly the same set up except for the CO_2_ enrichment. We considered that the perils of possible artifacts derived from pseudoreplication are small compared with the probability of Type II error associated with the error introduced when attempting at controlling CO_2_ independently in each replicated treatment. The plants were exposed to the experimental CO_2_ levels for 5 months (from March to August).

### Seawater chemistry

The daily fluctuations of dissolved inorganic carbon (CO_2_, HCO_3_^−^, and CO_3_^2−^), pH, and total alkalinity of the seawater in both CO_2_ treatments were monitored throughout the experiment at different hours during the day. In July, a complete 24 h cycle was made to illustrate the diel variation in seawater carbon chemistry. Triplicate water samples were collected inside the seagrass canopy in each mesocosm replicate every 2 h. For each replicate sample, total alkalinity was determined by measuring pH directly (Multimeter 340; WTW, Weilheim, Germany; accuracy of ±0.004 for the temperature range 15–35°C) in 4 mL of seawater before and after acidification with 1 mL of HCl 0.01 M, according to Parsons et al. (1984) and modified by Semesi et al. (2009). The concentration of dissolved inorganic carbon (CO_2_, HCO_3_^−^, and CO_3_^2−^) was calculated from total alkalinity, temperature, and salinity of the seawater using the Excel-based program CO_2_SYS.XLS 1.0 (Pelletier et al. 1997). Salinity was measured using a hand refractometer, whereas temperature was measured using a combined pH + temperature probe (SenTixHWS; WTW). Water samples for nutrient analysis were also collected in triplicate, filtered through cellulose acetate filters, and stored at −20°C. The concentrations of ammonium, nitrate, and phosphate in the seawater were determined in a loop-flow analyzer (*μ*Mac-1000; Systea, Anagni, Italy). Ammonium concentration was determined using the hypochlorite method and nitrate concentration was determined using the Cd-Cu column reduction method. Phosphate was determined using the molybdate and ascorbic acid colorimetric method.

### Photosynthetic measurements

All photosynthetic measurements were performed on the second youngest leaf of *Z. noltii* shoots. Electron transport rates (ETR) of *Z. noltii* plants exposed to both CO_2_ levels (360 and 700 ppm) were measured in vivo along 1 day in June, using a submersible pulse amplitude modulated (PAM) fluorometer (Diving-PAM; Heinz Walz, Effeltrich, Germany). Ambient light was measured simultaneously with the Diving-PAM external quantum sensor. ETR (*μ*mol e^−^/m^2^/s) was calculated using the equation ETR = Y × *I* × AF × 0.5, where *I* is irradiance (*μ*mol photon/m^2^/s), AF is the absorption factor, that is, the fraction of incident photosynthetic photon flux absorbed by the leaves, and 0.5 is the assumed proportion of photons absorbed by pigments associated with each photosystem. We acknowledge that intraspecific AF values can vary with geographic location, time of year, depth, leaf age, and nitrogen status. However, in our study, ETR were accessed at the same time for each point along the day in leaves of the same age with a similar previous light history. Therefore, we used a previously determined absorption factor (0.79 ± 0.02, *n* = 10; Silva and Santos 2003). The effective quantum yield of photosystem II (Y) was calculated using the equation (F′_m_−F_s_)/F′_m_, where F_s_ is the fluorescence in the light when only part of the reaction centers are closed and F_m_′ is the maximal fluorescence of a light adapted leaf immediately after closure of all reaction centers obtained through the application of a saturating light pulse (Genty et al. 1989).

Light response curves were determined in the laboratory by following oxygen evolution in square section incubation chambers (15 mL) coupled to a Clark-type oxygen electrode (DW3/CB1; Hansatech, Norfolk, U.K.). Actinic light was provided by a slide projector (Pradovit 150; Leica, Solms, Germany) equipped with a halogen lamp (Xenophot 150W; Osram, MüFCnchen, Germany). Ten light intensities (between 0 and 875 *μ*mol quanta/m^2^/s) were achieved using a series of neutral density filters. For both CO_2_ concentrations (360 and 700 ppm), GF/F filtered seawater from the respective treatment was used as incubation medium. For each replicate measurement (*n* = 4 for the current CO_2_ and *n* = 3 for the enriched CO_2_ concentration), two independent segments (≍2 cm long) of *Z. noltii* leaves were held vertically inside the chamber. During the measurements, the water in the incubation chamber was continuously stirred and the temperature was kept constant at 20°C. Each light step took approximately 7 min at steady-state photosynthesis, and the water in the reaction chambers was replaced by new water before each light step, to prevent experimental artifacts (Silva and Santos 2003). After each light curve, the area of the leaf segments was measured (122–148 mm^2^) and leaf tissues were dried at 60°C for 24 h. The adapted hyperbolic tangent model equation of Jassby and Platt (1976) was fitted to the net photosynthesis versus irradiance data plots:


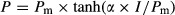


where P_m_ is the maximum photosynthetic rate (*μ*mol O_2_/m^2^/s), *I* is irradiance (*μ*mol quanta/m^2^/s), and *α* is the ascending slope at limiting irradiances (*μ*mol O_2_/*μ*mol quanta).

### Growth measurements

Leaf growth rates were determined in *Z. noltii* plants exposed to the two CO_2_ levels (360 and 700 ppm) at the end of the experiment using the classical punching method described for seagrasses by Zieman (1974) and modified by Peralta et al. (2000). For each CO_2_ level, the leaves of five random shoots in each mesocosm replicate unit were marked with fine plastic fibers immediately above the leaf sheath. The total length of nonmarked leaves (small or new leaves) was also recorded. After 3 days, the length from the leaf base to the punching mark and the total leaf length of nonmarked leaves were recorded. Leaf growth rate (LGR) (cm/d/shoot) was calculated following the equation:


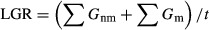


where G_nm_ is the growth rate of nonmarked leaves, G_m_ is the growth rate of marked leaves, and t is the time elapsed (days) between the punching and the final measurements (t_f_–t_0_). G_nm_ (cm/d/shoot) = TLL_f_–TLL_i_, where TLL_i_ and TLL_f_ are the total leaf length at t_0_ and t_f_, respectively. G_m_ (cm/d/shoot) = MLL_f_−MLL_i_, where MLL_i_ and MLL_f_ is the length from the leaf base to the punching mark at t_0_ and t_f_, respectively.

### Combined effect of nitrogen and CO_2_ concentration on nitrogen uptake rates

Leaf nitrogen uptake rates were estimated at the end of the experiment using two-compartment cylindrical chambers that physically separated the leaves from the belowground plant parts. Leakage between compartments was avoided using molding clay and sterile vaseline as sealants. The leaves of plants grown at 360 and 700 ppm CO_2_ were simultaneously incubated for 2 h in seawater enriched with ^15^NH_4_Cl or ^15^KNO_3_ solutions (atom% = 99; Cambridge Isotope Laboratories, Andover, MA) in a walk-in culture chamber at constant temperature (21°C) and light intensity (200 *μ*mol quanta/m^2^/s). The seawater used in the incubations was collected from the respective CO_2_ treatment batch. The uptake rates were determined at two nitrogen concentrations, one concentration that was representative of the typical values in the lagoon (5 *μ*M) and at another which represented a nutrient enriched scenario (30 *μ*M). This concentration is common in Ria Formosa in the vicinity of waste water treatment plants (Cabaço et al. 2008). Incubations at different CO_2_ levels were performed simultaneously for each nitrogen concentration, and replicate incubations (*n* = 3) were performed sequentially. One single shoot with the respective rhizome and roots was placed inside each split chamber. In the leaf compartment, an average leaf biomass of 0.04 g dry weight was incubated in 1.5 L of seawater, constantly mixed with a flow rate of ≍250 mL/min by a peristaltic pump. The nitrogen concentration (ammonium or nitrate) in the media did not vary noticeably throughout the incubation period. Root compartments were left without nutrients. Even though in natural conditions the rhizosphere of *Z. noltii* is mostly anoxic, in these experiments we incubated the whole plants in a nonanoxic medium. Previous experiments reported elsewhere (Alexandre et al. 2010) showed no effects of rhizosphere oxygenation on the ammonium and nitrate uptake rates of leaves.

At the end of incubations, the plants were removed from the chambers, the leaves were immediately separated from the rhizomes and roots and were briefly rinsed with deionized water to remove adherent label. Leaf tissues were dried at 60°C for 48 h and reduced to a fine powder. Total nitrogen content and atom% ^15^N of dried tissues were determined using a PDZ Europa ANCA-GSL elemental analyzer interfaced to a PDZ Europa 20–20 isotope ratio mass spectrometer (UC Davis, Davis, CA). Leaf ^15^N background levels were measured in three replicate samples.

### Nitrate reductase and glutamine synthetase activity

The incubations described in the previous section were repeated for the determination of nitrate reductase (NR) and glutamine synthetase (GS) activity. In these incubations, the leaf media were enriched with 30 *μ*M of nonlabeled NH_4_Cl and KNO_3_. The leaf incubations at different CO_2_ level were performed simultaneously, whereas replicate incubations (*n* = 3) were performed sequentially. NR activity was measured in vivo using the method described by Corzo and Niell (1991), optimized for *Z. noltii* (Alexandre et al. 2004). This method is based on the colorimetric measure of nitrite, formed after the reduction in nitrate by NR. Leaf tissue (0.12 g fresh weight) was incubated in 50 mM KNO_3_, 0.1M K_2_HPO_4_ (pH 8.0), 0.5 mM EDTA, and 0.5% 1- propanol, in a final assay medium volume of 10 mL, flushed with N_2_ for 2 min to remove oxygen. Incubations lasted 30 min at 30°C. The nitrite produced was measured spectrophotometrically (540 nm) after adding 1 mL of sulfanilamide and 1 mL of naphtyl-etylenediamine to the assay medium. The in vivo assay was chosen because it yielded consistently higher activity than the in vitro assay, which also failed to provide reproducible results (see also Touchette and Burkholder 2007 and references therein). GS activity was measured in vitro, using the method described by Sagi et al. (2002), optimized for *Z. noltii*. The normal biologic activity of GS combines ammonium with glutamate to yield glutamine. This reaction is mimicked in the synthetase assay, in which hydroxylamine is substituted for ammonium to yield the product γ-glutamyl-hydroxamate, which can be quantitated spectrophotometrically. Leaf tissue samples (0.12 g FW) were extracted in 1.6 mL of buffer containing 200 mM Tris buffer (pH 7.8), 2 mM EDTA, 3 mM dithiothreitol (DTT), 10 *μ*M flavin adenine dinucleotide (FAD), 10 mM MgCl, 2% (w/v) casein, 10% (v/v) glycerol, and 0.1 g polyvinylpyrrolidone (PVP). The homogenized plant material was centrifuged at 30,000 *g*, at 4°C for 15 min. A quantity of 100 *μ*L of the enzyme extract was added to 250 *μ*L of assay medium containing 18 mM ATP, 45 mM MgCl_2_·6H_2_O, 25 mM hydroxylamine, 92 mM l-glutamate, and 50 mM imidazole HCl (pH 7.2), at 30°C. After 20 min, the reaction was stopped by the addition of 0.5 mL of ferric chloride reagent (0.37 M ferric chloride, 0.67 M HCl, and 0.2 M trichloroacetic acid). The reaction solution was then centrifuged, and the absorbance of the supernatant was read at 540 nm.

### Data analysis

The effects of CO_2_ enrichment on net photosynthesis, leaf growth rates, and enzymatic activity were tested using *t*-tests. Differences in electron transport rates between CO_2_ levels were tested using Mann–Whitney nonparametric test because data were not normally distributed, even after transformation. Two-way analysis of variance was used to test significant effects of CO_2_ level and nutrient concentration on the ammonium uptake rates. A nonparametric Mann–Whitney test was used to detect significant effects of CO_2_ level on the nitrate uptake rate for each nutrient concentration. Effects were considered statistically significant at a level of *P* = 0.05.

## Results

### Seawater chemistry

**Figure 1:**
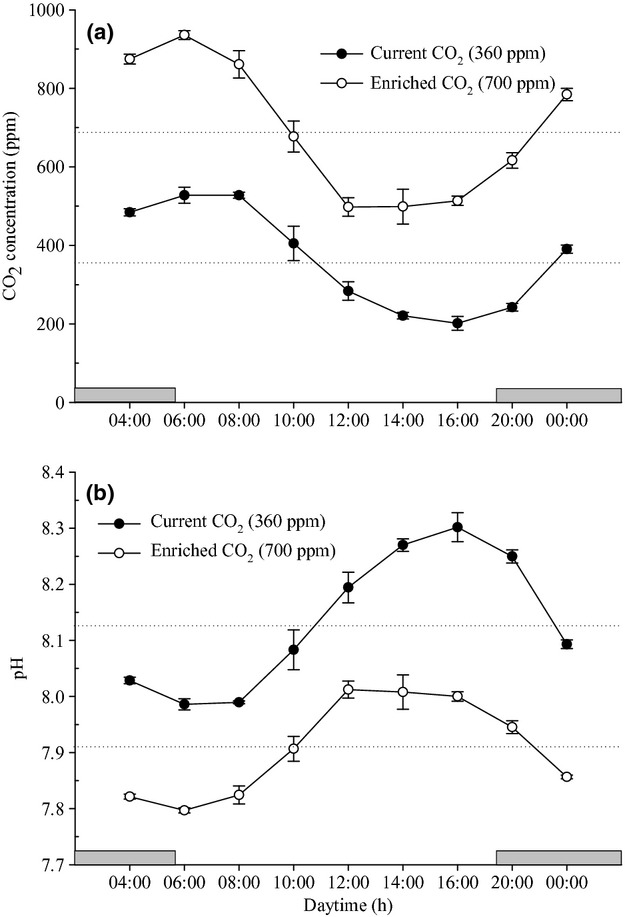
Daily fluctuation of (a) CO_2_ concentration (ppm) and (b) pH of the seawater in the control (open circle) and the CO_2_-enriched (closed circle) treatments. Dark areas represent nighttime. Values are mean ± SD (*n* = 6).

The daily fluctuation in the CO2 level and pH in the mesocosm was similar in both CO_2_ treatments (Fig. 1). CO_2_ increased during the night to a maximum at dawn decreasing throughout the day, whereas pH showed the opposite pattern. The concentration of CO_2_ and pH in the control-CO_2_ treatment averaged 360 ± 128 ppm and 8.13 ± 0.12, whereas in the enriched-CO_2_ treatment it averaged 695 ± 167 ppm and 7.91 ± 0.08, respectively.

As a consequence of CO_2_ addition to the system, the concentration of CO_2_ and HCO_3_^−^ increased in the enriched-CO_2_ treatment compared with the control, whereas the concentration of CO_3_^2−^ was reduced (Table 1). Total alkalinity was not significantly different between treatments and did not vary much along the day. The concentration of ammonium and nitrate in both treatments was nearly undetectable throughout the daily cycle (<0.01 *μ*M) suggesting that all the available inorganic nitrogen was being taken up by the plants. Phosphate concentration in the control and CO_2_-enriched treatment averaged 0.18 ± 0.03 and 0.24 ± 0.04 *μ*M, respectively.

**Table 1:** Daily fluctuation of the seawater carbonate speciation in the two experimental CO_2_ levels (360 and 700 ppm). Values of total carbon (TC), bicarbonate (HCO_3_^−^), and carbonate (CO_3_^2−^) were calculated using total alkalinity (TA), pH, salinity, and temperature of the seawater (Pelletier et al. 1997). Values are mean ± SD (*n* = 6) and represent pooled data from the two replicate mesocosm units. Units are *μ*mol/Kg

	360 ppm				700 ppm			
		
Daytime (h)	TA	TC	HCO_3_^−^	CO_3_^2−^	TA	TC	HCO_3_^−^	CO_3_^2−^
04:00	2722 ± 17	2340 ± 16	2040 ± 15	285 ± 3	2760 ± 18	2510 ± 17	2288 ± 16	198 ± 2
06:00	2629 ± 34	2278 ± 40	2015 ± 36	255 ± 3	2765 ± 14	2529 ± 13	2314 ± 12	190 ± 2
08:00	2656 ± 21	2295 ± 13	2032 ± 19	259 ± 2	2738 ± 24	2488 ± 20	2266 ± 20	198 ± 7
10:00	2661 ± 19	2260 ± 39	1926 ± 54	305 ± 17	2697 ± 10	2398 ± 9	2153 ± 17	227 ± 10
12:00	2653 ± 9	2121 ± 24	1730 ± 36	376 ± 16	2697 ± 15	2300 ± 22	2001 ± 26	286 ± 7
14:00	2622 ± 14	2035 ± 12	1613 ± 20	411 ± 6	2660 ± 16	2277 ± 24	1986 ± 35	277 ± 15
16:00	2627 ± 15	2028 ± 29	1582 ± 45	428 ± 14	2680 ± 14	2307 ± 13	2020 ± 13	273 ± 5
19:30	2702 ± 25	2145 ± 20	1695 ± 57	400 ± 18	2743 ± 29	2409 ± 26	2141 ± 24	251 ± 6
00:00	2743 ± 15	2238 ± 4	1878 ± 17	360 ± 5	2774 ± 42	2456 ± 39	2192 ± 35	244 ± 4

### Photosynthesis and growth

**Figure 2:**
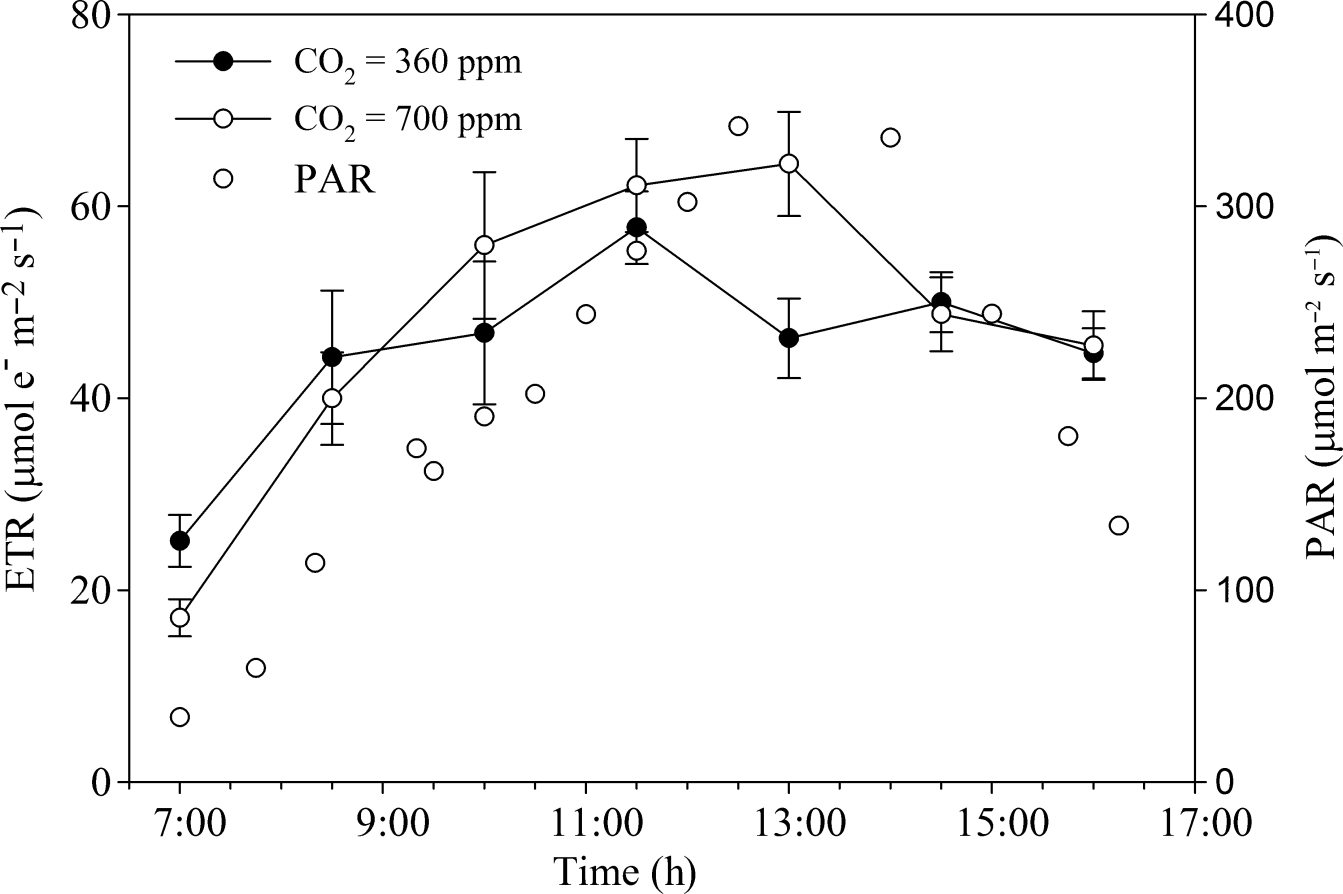
*Zostera noltii*. Diurnal variation in electron transport rate (ETR, *μ*mol e^−^/m^2^/s) and available photosynthetic active radiation (PAR) in plants exposed to current (360 ppm) and elevated (700 ppm) CO_2_ concentrations.

**Figure 3:**
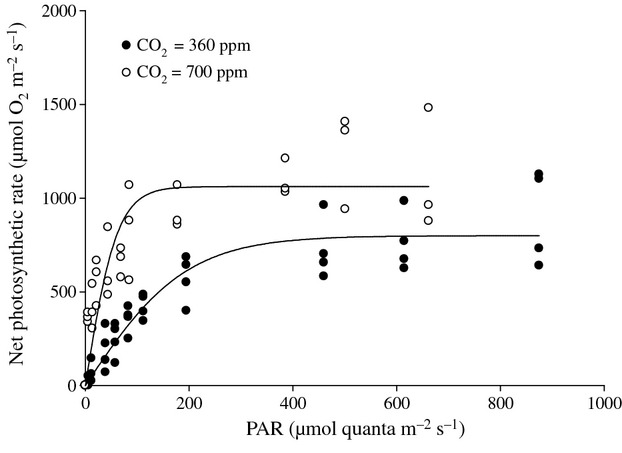
*Zostera noltii*. Net photosynthetic rate (*μ*mol O_2_/m^2^/s) versus photosynthetic active radiation (PAR; *μ*mol quanta/m^2^/s) measured following oxygen evolution determined at 20°C in leaf segments of plants exposed at 360 ppm (closed circles) and 700 ppm (open circles). Values are mean ± SD (*n* = 3–4).

The electron transport rates (ETR) of *Z. noltii* plants both under present and increased CO_2_ values, showed a typical variation, increasing during the morning, peaking at midday, and decreasing in the afternoon (Fig. 2). At peak irradiance values (13:00, PAR ≍ 300 *μ*mol/m^2^/s), the ETR of control plants showed a sharp decline that suggests a dynamic downregulation of photosynthesis, whereas the CO_2_-enriched plants did not. However, the whole ETR values of control plants were not significantly different from those of plants exposed to CO_2_-enriched conditions (*P* = 0.39) (Fig. 2). The irradiance-saturated photosynthetic rate (P_m_) of plants exposed to CO_2_-enriched conditions (1061.5 ± 60.5 *μ*mol O_2_/m^2^/s) was 1.3-fold higher than the rate of plants exposed to current CO_2_ concentration (799.4 ± 36.2 *μ*mol O_2_/m^2^/s) (Fig. 3). Similarly, the photosynthetic rates at limiting irradiances (*α*), expressed as photosynthetic efficiency, were much higher in CO_2_-enriched plants (17.3 ± 2.7 *μ*mol O_2_/*μ*mol quanta) than in plants exposed to current CO_2_ concentration (4.1 ± 0.4 *μ*mol O_2_/*μ*mol quanta). On the other hand, no significant effect of CO_2_ enrichment was detected on the leaf growth rate of *Z. noltii*. The leaf growth rate of plants exposed to elevated CO_2_ concentration was 1.12 ± 0.27 cm/d/shoot, whereas the rate of plants grown at current CO_2_ conditions was 1.18 ± 0.21 cm/d/shoot.

### Nitrogen uptake

**Figure 4:**
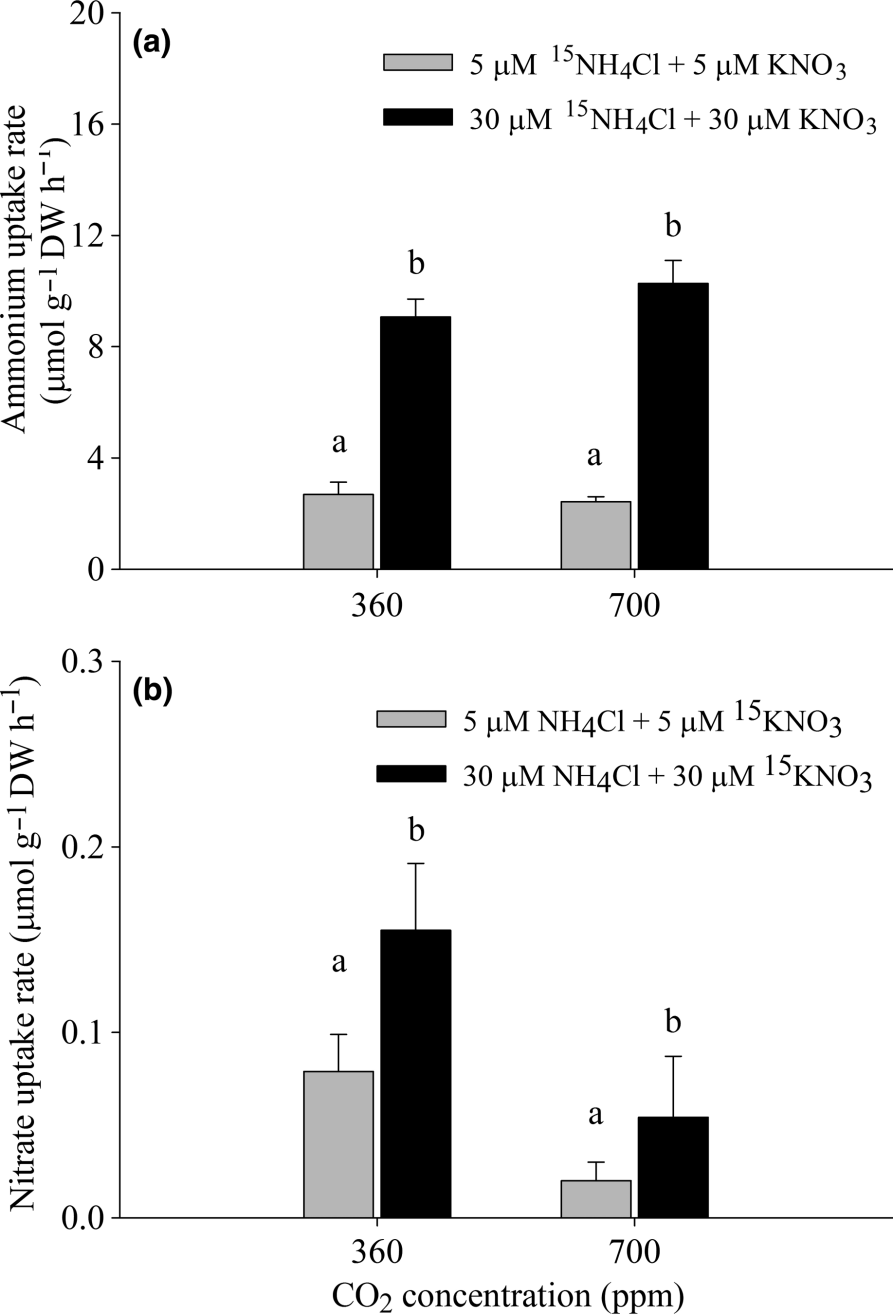
*Zostera noltii*. Ammonium (a) and nitrate (b) uptake rates (*μ*mol/g DW/h) of plants leaves exposed to CO_2_ concentrations of 360 and 700 ppm when incubated at 5 and 30 *μ*M of NH_4_Cl + KNO_3_. Values are mean ± SE (*n* = 6). Different letters denote significant differences.

The ammonium uptake rates of leaves exposed to higher CO_2_ concentration, either incubated with 5 *μ*M (2.42 ± 0.18 *μ*mol/g DW/h^−1^) or 30 *μ*M ^15^NH_4_Cl (10.27 ± 0.83 *μ*mol/g DW/h), were not significantly different from the rates of plants exposed to the current CO_2_ conditions (2.69 ± 0.44 and 9.07 ± 0.64 *μ*mol/g DW/h, respectively, *P* = 0.427) (Fig. 4, Table 2). Similarly, both the nitrate uptake rates of CO_2_-enriched leaves incubated at 5 *μ*M (0.02 ± 0.01 *μ*mol/g DW/h) and at 30 *μ*M (0.05 ± 0.03 *μ*mol/g DW/h) were not significantly different from the control (0.08 ± 0.02 *μ*mol/g DW/h, *P* = 0.065, and 0.16 ± 0.04 *μ*mol/g DW/h, *P* = 0.240, respectively), but the leaf nitrate uptake rate of plants exposed to CO_2_-enriched conditions was fourfold lower than the uptake of plants exposed to current CO_2_ level.

**Table 2:** Combined effects of CO_2_ and dissolved inorganic nitrogen on the ammonium uptake rates of *Zostera noltii*, as determined by two-way analysis of variance

	df	MS	*F*	*P*
Ammonium uptake				
CO_2_	1	1.307	0.658	0.427
N concentration	1	303.599	152.85	<0.001
CO_2_ × N concentration	1	3.229	1.626	0.217

### Enzymatic activity

**Figure 5:**
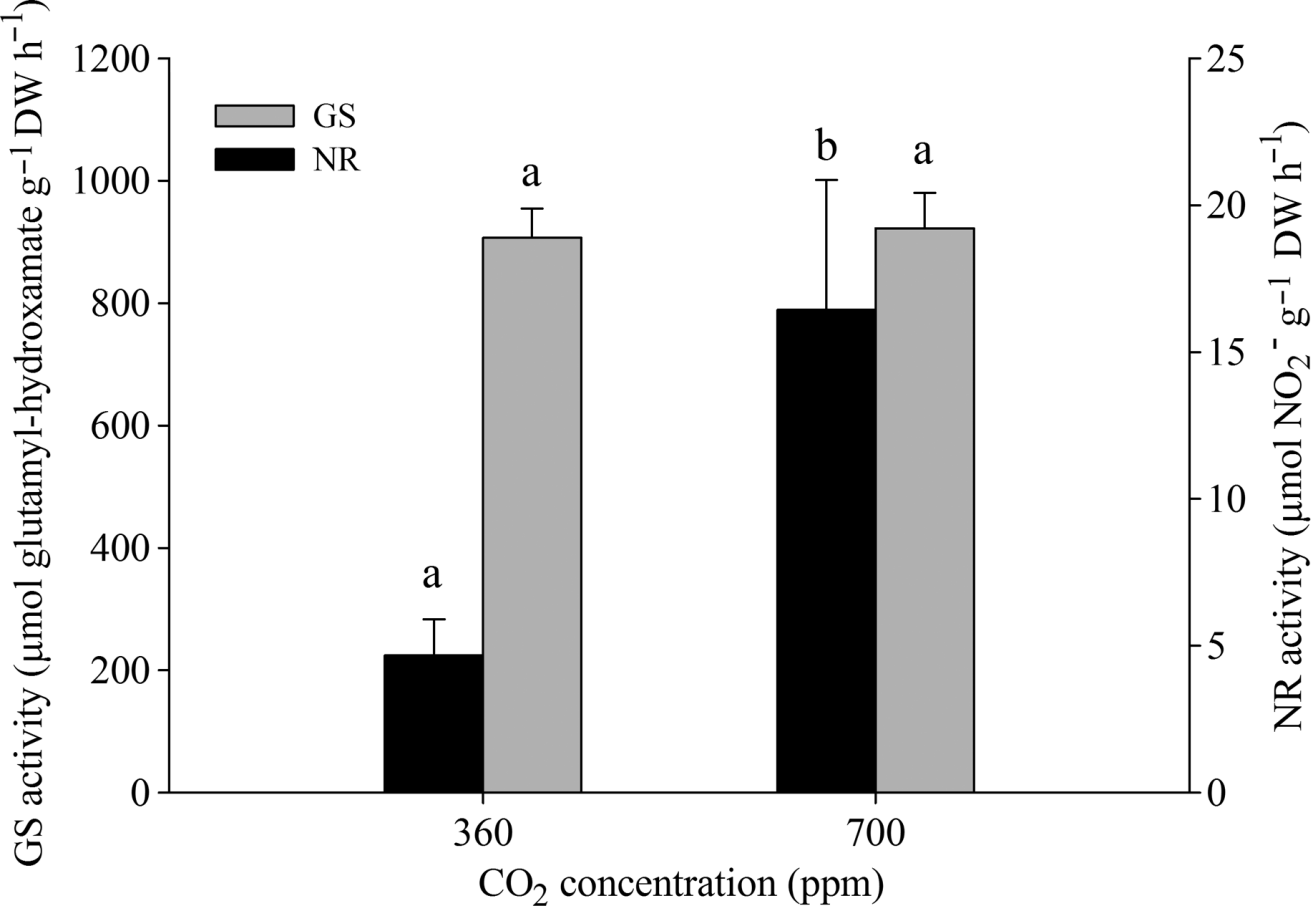
*Zostera noltii*. Effect of CO_2_ enrichment on the activity of the enzymes glutamine synthetase (GS) (gray bars) and nitrate reductase (NR) (black bars). Values are mean ± SE (*n* = 3). Different letters denote significant differences.

The activity of the enzyme glutamine synthetase of plant leaves grown under CO_2_ enrichment (923 ± 58 *μ*mol glutamil-hydroxamate/g DW/h) was not significantly different from that of plants from the control treatment (907 ± 48 *μ*mol glutamil-hydroxamate/g DW/h) (Fig. 5). On the other hand, the activity of nitrate reductase was threefold higher (16.4 ± 4.4 *μ*mol NO_2_^−^/g DW/h) in the leaves of plants exposed to higher CO_2_ concentration than in control plants (4.7 ± 1.2 *μ*mol NO_2_^−^/g DW/h).

## Discussion

This study showed that the net photosynthetic rate of *Z. noltii* is positively affected by the CO_2_ enrichment of the seawater. Plants exposed to CO_2_-enriched conditions showed higher photosynthetic rates at saturating irradiances and were photosynthetically more efficient at limiting light intensities (higher *α*) when compared with plants exposed to the current CO_2_ concentration. This was probably the result of a higher carboxylation activity relatively to oxygenation activity from RuBisCO in the presence of a higher CO_2_/O_2_ ratio, as CO_2_ and O_2_ are competitive inhibitors for RuBisCO's active site (Taiz and Zeiger 2002). The higher carboxylation activity relatively to oxygenation activity results in lower photorespiration, which decreases the energy cost for CO_2_ fixation and increases the photochemical quantum yield (Furbank 1998; Taiz and Zeiger 2002). CO_2_-enriched plants showed higher ETR values at peak light intensity contrasting with the control plants that showed much lower values, suggesting a dynamic downregulation of photosynthesis. This indicates that photosynthesis of *Z. noltii* is C_i_-limited at the current inorganic carbon concentration of seawater, confirming the conclusions previously obtained for the same species (Silva et al. 2005), and for other seagrass species (Beer and Koch 1996; Zimmerman et al. 1997; Invers et al. 2001). These results also suggest that *Z. noltii* may benefit from future CO_2_ enrichment by enhancing the photosynthetic rates at higher CO_2_ concentrations. The CO_2_-stimulated increase in photosynthesis found here for *Z. noltii* is consistent with the findings reported for the temperate and tropical seagrass species *Z. marina* and *Thalassia hemprichii*, where positive photosynthetic responses to CO_2_ enrichment were also found (Beer and Koch 1996; Thom 1996; Zimmerman et al. 1997; Jiang et al. 2010).

Our results showed that CO_2_ enrichment did not stimulate the growth rate of *Z. noltii* leaves exposed to increased CO_2_ for 5 months. This finding is consistent with Palacios and Zimmerman (2007) observations for *Z. marina*, but contrary to Thom (1996) and Jiang et al. (2010) studies, where CO_2_ enrichment enhanced leaf growth rates of *Z. marina* and *T. hemprichii*, respectively. However, CO_2_ enrichment did have an effect on belowground growth rates of *Z marina* (Palacios and Zimmerman 2007). Observations of enhanced seaweed growth rates under high-CO_2_ levels have also been reported (Gao et al. 1991, 1993; Gordillo et al. 2001; Zou 2005; Xu et al. 2010). However, these studies investigated the effects of CO_2_ enrichment using only short-term experiments (days). Longer term experiments (months) with seagrass species found no significant differences in leaf growth rates of plants exposed to current and elevated CO_2_ concentrations (Palacios and Zimmerman 2007; this study). In some terrestrial plant species short-term exposure to elevated CO_2_ resulted in decreased photorespiration and even inhibition of dark respiration (Amthor 1991; Cousins et al. 2001). In the long term, the inhibition of the respiratory metabolism may result in a decrease in starch catabolism and available energy, with possible consequences for plant growth (Yelle et al. 1990). On the other hand, there is evidence that the stimulation of growth by elevated CO_2_ concentrations can be strongly curtailed in plants grown under nitrogen-limited conditions (Stitt and Krapp 1999 and references therein; Liu et al. 2010). Nitrogen may thus become the limiting factor of plant production in the enriched CO_2_ future. This was previously reported for the seaweed *Ulva* sp., which more than doubled its growth rate when cultivated at CO_2_-enriched conditions, whereas under nitrogen-limited conditions its growth was only slightly increased (Gordillo et al. 2001). The nitrogen status of the plants, which is a consequence of the nitrogen growth conditions, has been used to determine the expression of CO_2_ enrichment effects on growth rates (Andría et al. 1999; Gordillo et al. 2001). The leaf nitrogen content of *Z. noltii* plants from both experimental CO_2_ treatments (1.4%) was below the critical level of 1.8% reported as indicative of low nitrogen supply (Duarte 1990), suggesting that the nitrogen available for the plants in the mesocosm (the natural concentration available in Ria Formosa lagoon) was insufficient to fully meet the species nitrogen requirements for growth. Therefore, we hypothesize that the growth rates of *Z. noltii* plants were primarily controlled by the low nitrogen availability rather than by the elevated CO_2_ concentration in the mesocosm. An important corollary of this is that the global effects of CO_2_ on seagrass growth may not be spatially homogeneous and will depend on the specific availability of nitrogen, as well as other nutrients, such as P and Fe, of each system. Under conditions of elevated CO_2_ and nitrogen limitation, it is possible that the additional fixed C from higher photosynthetic rates remains stored until nitrogen availability is restored to levels that meet the nitrogen requirements for growth. On the other hand, there is the possibility that the additional fixed carbon is exudated in the form of DOC, or that it was used for belowground growth and shoot proliferation (Palacios and Zimmerman 2007), which were not assessed in this study.

Surprisingly, there were no significant effects of CO_2_ enrichment on the nitrate and ammonium uptake rates. These findings do not corroborate our initial hypothesis that the lower pH of CO_2_-enriched seawater would increase the nitrate uptake rates because in higher plants nitrate is cotransported along with H^+^ through the membrane (Vessey et al. 1990) and that the ammonium uptake rates would decrease, as the activity of H^+^-ATPase (involved in the cation transport into the cells) is reduced by the higher H^+^ content (Marschner 1995). Research on the nitrate transport system of *Z. marina* leaves (García-Sánchez et al. 2000) suggested that nitrate uptake in this species is probably not coupled with H^+^, as it is in other angiosperms (Ullrich 1992). A similar situation may occur in *Z. noltii*.

Factors other than pH might be involved in the decrease in the nitrate uptake rates of *Z. noltii* observed at high CO_2_. A nitrogen-limited seaweed *Ulva lactuca* also showed much lower nitrate uptake rates when exposed to elevated CO_2_ conditions (Magnusson et al. 1996). The authors concluded that the decreasing effect of CO_2_ enrichment on the nitrate uptake rates was not related to the pH level of the seawater and suggested that uncontrolled CO_2_ entrance in the cellular compartments may affect regulatory mechanisms and enzyme functioning with consequences for the nutrient uptake rates.

The nitrate assimilatory capacity of *Z. noltii* was positively affected by the CO_2_ enrichment, as revealed by the higher nitrate reductase activity of plant leaves grown under CO_2_-enriched conditions, while nitrate uptake rate was reduced. These results indicate that nitrate uptake and reduction are uncoupled when *Z. noltii* is grown at high CO_2_. Some alteration in the production of ATP relative to NADPH might also explain the imbalance between nitrate reductase activity and assimilation of nitrogen found in *Z. noltii* grown at high-CO_2_ conditions, as suggested by Mercado et al. (1999). The CO_2_-driven stimulation of this enzyme's activity was also reported for terrestrial plants and seaweeds (Fonseca et al. 1997; Mercado et al. 1999; Gordillo et al. 2001; Zou 2005). In terrestrial plants, it has been suggested that elevated CO_2_ controls nitrate assimilation indirectly through the amount of accumulated carbohydrates (Fonseca et al. 1997). Increased accumulation of carbohydrates, such as soluble sugars and starch, has been observed in both seagrass and seaweed species grown at elevated CO_2_ concentrations as a consequence of limiting nitrogen regimes (Zimmerman et al. 1995, 1997; Andría et al. 1999; Jiang et al. 2010). Under nitrogen limitation, the increased photosynthetic activity observed in *Z. noltii* may have caused an imbalance between the carbon supply and its utilization for growth, leading to an accumulation of carbohydrates. We hypothesize that *Z. noltii* plants exposed to elevated-CO_2_ concentrations may have accumulated higher levels of carbohydrates, which contributed to increase the nitrate reductase activity by supplying energy and carbon skeletons for the nitrate reduction process. On the other hand, it has also been suggested that the CO_2_-driven increase in the maximum nitrate reductase activity is not regulated by the carbohydrate level or internal carbon content but rather through a direct action on the enzyme synthesis, which is triggered by nitrate signaling (Gordillo et al. 2001). This is an interesting topic that deserves further investigation.

In conclusion, the photosynthetic rate of *Z. noltii* increased under high-CO_2_ conditions, but no effects were detected on growth probably because plants were nutrient limited, as revealed by the low total nitrogen content of the plants at the end of the experiment. Under a CO_2_ increase scenario, the natural levels of nutrients will probably become limiting for *Z. noltii*. This potential limitation becomes more relevant because the expected positive effect of CO_2_ increase on nitrate uptake rate was not confirmed.
