# Corrigendum

**DOI:** 10.1002/ece3.1798

**Published:** 2015-10-26

Ana Alexandre, João Silva, Pimchanok Buapet, Mats Bjork & Rui Santos. Effects of CO_2_ enrichment on photosynthesis, growth, and nitrogen metabolism of the seagrass *Zostera noltii*, Ecology and Evolution 2012; 2(10), 2620–2630. DOI: 10.1002/ece3.333.

In the above‐mentioned article, the values of the net photosynthetic rate presented in Figure 3 were incorrectly calculated during the conversion from *μ*mol O_2_ g^−1^ FW h^−1^ into *μ*mol O_2_ m^−2^ s^−1^, resulting in their overestimation. Below is the corrected Figure 3.

**Figure 3:**
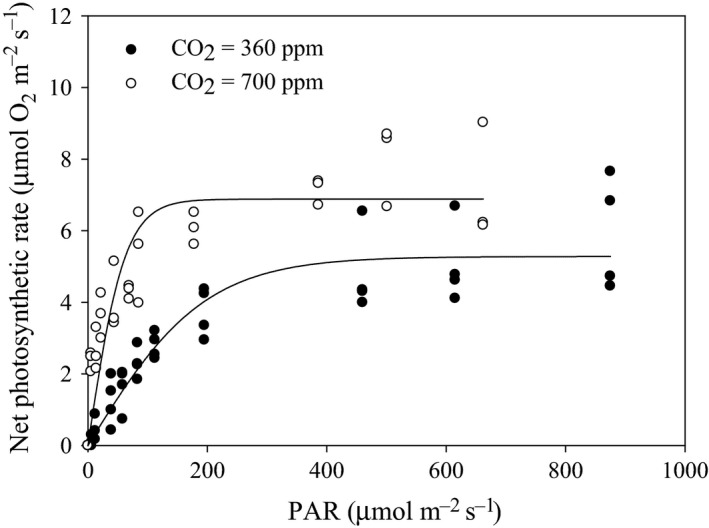
*Zostera noltii*. Net photosynthetic rate (*μ*mol O_2_/m^2^/s) versus photosynthetic active radiation (PAR;* μ*mol quanta/m^2^/s) measured following oxygen evolution determined at 20°C in leaf segments of plants exposed at 360 ppm (closed circles) and 700 ppm (open circles). Values are mean ± SD (*n* = 3–4).

The values of P_max_ and *α* extracted from the two PI curves in Figure 3 need also correction. The text on page 6 should be replaced by “The irradiance‐saturated photosynthetic rate (P_m_) of plants exposed to CO_2_‐enriched conditions (6.88 ± 0.35 *μ*mol O_2_/m^2^/s) was 1.3‐fold higher than the rate of plants exposed to current CO_2_ concentration (5.28 ± 0.23 *μ*mol O_2_/m^2^/s) (Fig. 3). Similarly, the photosynthetic rates at limiting irradiances (*α*), expressed as photosynthetic efficiency, were much higher in CO_2_‐enriched plants (0.11 ± 0.02 *μ*mol O_2_/*μ*mol quanta) than in plants exposed to current CO_2_ concentration (0.03 ± 0.003 *μ*mol O_2_/*μ*mol quanta).”

The new photosynthetic values do not change the interpretation of the results and the overall conclusions of the paper remain unchanged.

